# Supplementary material for: Systematic analysis of genetic and phenotypic characteristics reveals antisense oligonucleotide therapy potential for one-third of neurodevelopmental disorders
Source: Genome Med. 2025 May 21;17:59. doi: 10.1186/s13073-025-01477-x (PMC12096787; doi:10.1186/s13073-025-01477-x)
Supplement: Supplementary file 2 — Additional file 2: Fig. S1. Source score contribution per source NDD database. Table S1: Assumptions and variables per AON strategy. Table S2: Average number of amenable individuals per AONs per AON strategy. [file 13073_2025_1477_MOESM2_ESM.pdf]

Additional file 2

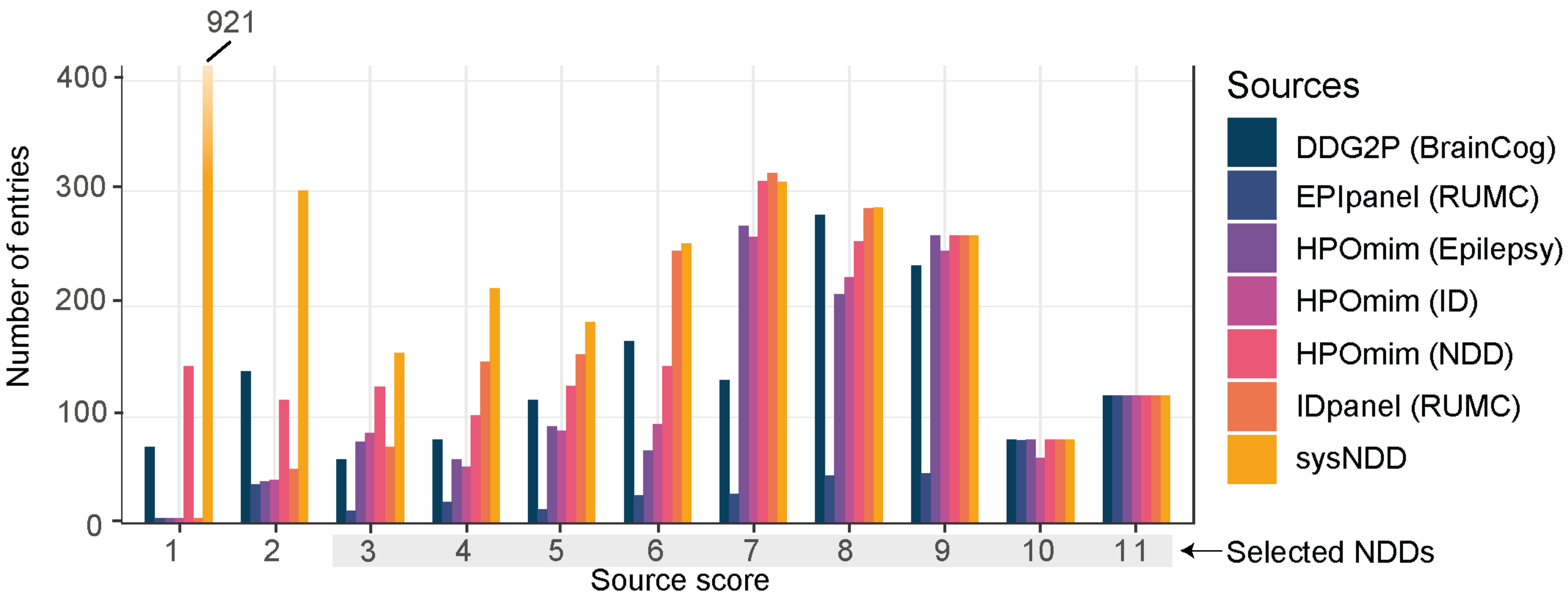

Fig. S1. Source score contribution per source NDD database. NDD: Neurdevelopmental disorder. The bars represent the contribution of the following databases to the source scores: sysNDD: European Reference Network for Rare Malformation Syndromes, Intellectual and Other Neurodevelopmental Disorders (ERN-ITHACA; sysNDD), Entries related to brain & cognition (BrainCog) of the Developmental Disorders Genotype-to-Phenotype (DDG2P, DDD) database from the UK Deciphering Developmental Disorders project (DDD), the Human Phenotype Ontology (HPO; HPOmim) intellectual disability (ID; HP_0001249), NDD (HP_0012758), Epilepsy (HP_0001250), in-house diagnostic gene-panel of the RadboudUMC (RUMC) for ID and epilepsy (EPIpanel, IDpanel). The NDDs that were selected for the targetability assessment (source score > 2) are highlighted in light grey.

Table S1. Assumptions and variables per AON strategy.

Strategy	Assumption*	Filter criteria/variable
**Splice correction**	Splice-correction is only feasible intronic splice-variants that do not affect canonical splice sites can be targeted with splice-correction AONs	VEP-consequence = Intronic variants, location = >50bp away from canonical splice sites (in the MANE transcript)
**ES-RF**	ES-RF is only feasible for fully deleted exons (leading to a frame shift) or splice-variants leading to skipping of an out-of-frame exon	Variant type = deletion (of an out-of-frame exon) or VEP-consequence = splice-donor/acceptor variants (flanking an out-of-frame exon)
	ES-RF is only achievable if skipping a single exon -immediately adjacent to the deletion or skipped out-of-frame exon - corrects the predicted frame shift caused by the mutation	Frame-shift restoration possible by skipping at least one adjacent exons
	ES-RF does not lead to a functional protein if the first or last exon is lost by either the mutation or due to the AON	Exon (deleted or skipped by the AON) must not be the first or last exon (in the MANE transcript)
	ES-RF does not lead to a functional protein if more than 10% of the protein is lost.	≤10% protein loss when mutations and skipped exon are considered.
**ES-VE**	ES-VE is only feasible if it does not affect the reading frame	Variant in an in-frame exon
	ES-VE does not lead to a functional protein if the first or last exon is skipped by the AON	Exon (skipped by the AON) must not be the first or last exon (in the MANE transcript)
	ES-VE is only applicable for stop-gained and frame-shift variants and not missense variants	VEP-consequence = splice-gained variant or frame-shift variant
	ES-VE does not lead to a functional protein if more than 10% of the protein is lost.	≤10% protein loss when mutations and skipped exon are considered.
**TANGO**	Any detectable non-productive splice event is potentially targetable for TANGO	1 or more non-productive splice event in the affected gene
	TANGO requires the variant to lead to loss of function	pLI > 0.9 & >20% LoF variants (VEP-consequence = stop-gained, frame-shift or splice-donor/acceptor variant)
	TANGO requires the presence of one intact allele to be upregulated	inheritance = AD
**uAUG-targeting**	Any detectable non-productive splice event is potentially targetable for uAUG-targeting	1 or more uAUG in the affected gene
	uAUG-targeting requires the variant to lead to loss of function	pLI > 0.9 & >20% LoF variants (VEP-consequence = stop-gained, frame-shift or splice-donor/acceptor variant)

	uAUG-targeting requires the presence of one intact allele to be upregulated	inheritance = AD
**ARE-targeting**	Any detectable non-productive splice event is potentially targetable for ARE-targeting	1 or more ARE in the affected gene
	ARE-targeting requires the variant to lead to loss of function	pLI > 0.9 & >20% LoF variants (VEP-consequence = stop-gained, frame-shift or splice-donor/acceptor variant)
	ARE-targeting requires the presence of one intact allele to be upregulated	inheritance = AD
**lncRNA-targeting**	A lncRNA should regulate gene-expression of the affected allele	1 or more lncRNA within the same TAD as the affected gene
		Correlation in expression between lncRNA and the affected gene in GTEX brain data
	lncRNA-targeting requires the variant to lead to loss of function	pLI > 0.9 & >20% LoF variants (VEP-consequence = stop-gained, frame-shift or splice-donor/acceptor variant)
	lncRNA-targeting requires the presence of one intact allele to be upregulated	inheritance = AD
**all strategies**	AON therapy is viable for patients if the phenotypic feature can be reversed or if the therapy has the potential to halt disease progression	Disorders link to HPO-terms (in OMIM): developmental, mental or ambulation deterioration (HP:0002376, HP:0001268, HP:0002505), neurodegeneration (HP:0007367), or seizures (HP:0001250) or abnormality of movement (HP:0100022)** or any HPO-term downstream of these HPO terms
	The time-window for AON therapy is too short to treat NDDs causing neonatal death	Disorders link to HPO-terms (in OMIM): neonatal death (HP:0003811) are excluded

AON: Antisense oligonucleotide, VEP: Variant Effect Predictor, ES-RF: Exon skipping for reading frame restoration , ES-VE: Exon skipping for variant exclusion, TANGO: Targeted Augmentation of Nuclear Gene Output, LoF: Loss of Function, AD: Autosomal Dominant, ARE: AU-rich element, lncRNA: long non-coding RNA, TAD: Topologically associated domain, HPO: Human Phenotype Ontology. **The assumptions presented in this table were made to facilitate analyses while realizing that true complexity of both therapeutic opportunities and/or phenotypic presentation and/or biological impact is underestimated by these assumptions. They should, as such, be interpreted as approximations based on the available data, tools and information and context. With increasing insights, the analyses can be repeated in our framework by adapting the underlying assumptions and filter criteria. **Of note, movement disorders do not typically have an etiology in the peripheral nervous system, but are thought to be generated by dysfunction of the deep brain nuclei and particularly the basal ganglia, making them a candidate phenotype to target.*

Table S2. Average number of amenable individuals per AONs per AON strategy

AON strategy	Number of submissions variants	Predicted number of AONs	Individuals per AON
**Splice-correction**	359	170	2
**Splice-correction of recurrent variants**	198	43	5
**ES-RF**	2744	810	3
**ES-VE**	10262	1738	6
**TANGO**	10499	91	115
**uAUG interference**	8305	69	120
**ARE inhibition**	4884	88	56
**lncRNA targeting**	11299	165	68

AON: Antisense oligonucleotide, ES-RF: Exon skipping for reading frame restoration, ES-VE: Exon skipping for variant exclusion, TANGO: Targeted Augmentation of Nuclear Gene Output, uAUG: upstream start codon, ARE: AU-rich element, lncRNA: long non-coding RNA.
